# Early Hepatic Dysfunction Is Associated with a Worse Outcome in Patients Presenting with Acute Respiratory Distress Syndrome: A Post-Hoc Analysis of the ACURASYS and PROSEVA Studies

**DOI:** 10.1371/journal.pone.0144278

**Published:** 2015-12-04

**Authors:** Stéphanie Dizier, Jean-Marie Forel, Louis Ayzac, Jean-Christophe Richard, Sami Hraiech, Samuel Lehingue, Anderson Loundou, Antoine Roch, Claude Guerin, Laurent Papazian, ACURASYS study investigators, PROSEVA Study Group

**Affiliations:** 1 Assistance Publique - Hôpitaux de Marseille, Hôpital Nord, Réanimation des Détresses Respiratoires et des Infections Sévères, 13015, Marseille, France; 2 Aix-Marseille Université, Faculté de médecine, URMITE UMR CNRS 7278, 13005, Marseille, France; 3 Hospices Civils de Lyon, Hôpital Henri Gabrielle, CClin Sud Est, 69230, Saint Genis Aval, France; 4 Hospices Civils de Lyon, Hôpital de la Croix-Rousse, Réanimation médicale et Surveillance Continue, 69004, Lyon, France; 5 Unité d'Aide Méthodologique à la Recherche clinique DRRC/AP-HM, Laboratoire de Santé Publique Faculté de Médecine, 13005, Marseille, France; Erasmus Medical Centre, NETHERLANDS

## Abstract

**Introduction:**

Bilirubin is well-recognized marker of hepatic dysfunction in intensive care unit (ICU) patients. Multiple organ failure often complicates acute respiratory distress syndrome (ARDS) evolution and is associated with high mortality. The effect of early hepatic dysfunction on ARDS mortality has been poorly investigated. We evaluated the incidence and the prognostic significance of increased serum bilirubin levels in the initial phase of ARDS.

**Methods:**

The data of 805 patients with ARDS were retrospectively analysed. This population was extracted from two recent multicenter, prospective and randomised trials. Patients presenting with ARDS with a ratio of the partial pressure of arterial oxygen to the fraction of inspired oxygen < 150 mmHg measured with a PEEP ≥ 5 cm of water were included. The total serum bilirubin was measured at inclusion and at days 2, 4, 7 and 14. The primary objective was to analyse the bilirubin at inclusion according to the 90-day mortality rate.

**Results:**

The 90-day mortality rate was 33.8% (n = 272). The non-survivors were older, had higher Sepsis-related Organ Failure Assessment (SOFA) score and were more likely to have a medical diagnosis on admission than the survivors. At inclusion, the SOFA score without the liver score (10.3±2.9 vs. 9.0±3.0, p<0.0001) and the serum bilirubin levels (36.1±57.0 vs. 20.5±31.5 μmol/L, p<0.0001) were significantly higher in the non-survivors than in the survivors. Age, the hepatic SOFA score, the coagulation SOFA score, the arterial pH level, and the plateau pressure were independently associated with 90-day mortality in patients with ARDS.

**Conclusion:**

Bilirubin used as a surrogate marker of hepatic dysfunction and measured early in the course of ARDS was associated with the 90-day mortality rate.

## Introduction

Over the last 20 years, our understanding of the risk factors and the mechanisms of acute lung injury has improved. However, acute respiratory distress syndrome (ARDS) is still associated with a high mortality rate, especially for the most severe forms of ARDS [1, 2]. Some strategies have been shown to improve the outcome, such as the use of a reduced tidal volume, the use of prone positioning [3] and when neuromuscular blocking agents (NMBA) [4] are administered to the most severely hypoxaemic patients. It is well-known that ARDS evolution is commonly associated with the progressive apparition of other organ failures. Mortality is finally mainly related to these associated organ failures whereas refractory hypoxaemia is uncommon in late deaths. Among these organ failures, hepatic dysfunction complicating ARDS has been poorly studied. It is generally acknowledged that such organ failure results from the association of hypoperfusion, hypoxaemia and passive congestion of the liver. Some studies have shown a relationship between hepatic dysfunction and poor outcome in critically ill patients [5–8]. Bilirubin is recognized as a stable and powerful marker of hepatic dysfunction and is used in scoring algorithms to assess the prognosis in critically ill patients and/or to predict the mortality risk in patients with ARDS [9–12]. Only few studies have been performed in ARDS patients regarding liver function. In a series of 88 patients, Fowler et al. [13] did not report a difference in mortality between the nine ARDS patients with liver failure and the 79 patients without liver failure. In a small series of 22 patients dying more than 72 hours after the onset of ARDS, a severe hepatic dysfunction was reported as an indirect cause of death in four of them [14]. In 1989, Schwartz et al. [15] reported that bilirubin was higher early in the course of ARDS in nonsurvivors than in survivors in a series of 24 patients. All these studies included less than 100 patients and, much more importantly, were performed prior to the lung protective mechanical ventilation strategy era. The main objective of this study was to investigate the prognostic significance of an early hepatic dysfunction assessed by bilirubin in a cohort of moderate to severe ARDS patients included in two randomized controlled trials in which a strict and well-defined lung protective mechanical ventilation strategy was used.

## Patients and Methods

### Patients

The data were extracted from two multicenter, prospective, randomized controlled studies that evaluated the effect of prone positioning and neuromuscular blockers on 90-day mortality rate in moderate/severe ARDS patients (PROSEVA [3] and ACURASYS [4]). Concerning the PROSEVA study (NCT00527813), the protocol was approved by the ethics committee Comité Consultatif de Protection des Personnes dans la Recherche Biomedicale Sud-Est IV in Lyon, France, and by the Clinical Investigation Ethics Committee at Hospital de Sant Pau in Barcelona. The ACURASYS study (NCT00299650) was approved by the ethics committee of the Marseille University Hospital (Comite Consultatif de Protection des Personnes dans la Recherche Biomedicale). As the analysis presented here was done retrospectively and according to French law, no supplemental ethical approval nor informed consent from the patients were needed. Patients’ records were anonymized and de-identified prior to analysis. A total of 805 patients presenting with ARDS were prospectively included in these two trials from March 2006 through July 2011 from 26 ICUs in France and 1 in Spain. The inclusion criteria were the same for the two trials regarding the definition of ARDS. Patients receiving invasive mechanical ventilation for ARDS for less than 48 hours were included if the ratio of the partial pressure of the arterial oxygen (PaO_2_) to the fraction of the inspired oxygen (FiO_2_) (P/F ratio) was less than 150 mmHg, evaluated with a PEEP of at least 5 cm H_2_O and a tidal volume of 6 to 8 ml per kilogram of predicted body weight. The non-inclusion criteria were age younger than 18 years, lack of consent, known pregnancy, enrollment in another trial within the previous 30 days, increased intracranial pressure, severe chronic respiratory disease requiring long-term oxygen therapy or mechanical ventilation at home, pneumothorax, and decision to withhold life-sustaining treatment. Specific non-inclusion criteria in the ACURASYS study [4] were continuous infusion of a neuromuscular blocking agent at enrollment, actual body weight exceeding 1 kg per centimeter of height, severe chronic liver disease (Child–Pugh class C), bone marrow transplantation or chemotherapy-induced neutropenia, expected duration of mechanical ventilation of less than 48 hours, and time window missed. Specific non-inclusion criteria in the PROSEVA study [3] were massive hemoptysis requiring an immediate surgical or interventional radiology procedure, tracheal surgery or sternotomy during the previous 15 days, serious facial trauma or facial surgery during the previous 15 days, deep venous thrombosis treated for less than 2 days, cardiac pacemaker inserted in the last 2 days, unstable spine, femur, or pelvic fractures, mean arterial pressure lower than 65 mm Hg, inhaled nitric oxide, almitrine bismesylate prone position or extracorporeal membrane oxygenation (ECMO) use before inclusion, lung transplantation, burns on more than 20% of the body surface, underlying disease with a life expectancy of less than one year, non-invasive ventilation delivered for more than 24 hours before inclusion, prone positioning before inclusion. As extensively stated in the original articles (PROSEVA [3] and ACURASYS [4]), a rigorous lung-protective strategy was applied in all of the patients, with well-defined and similar oxygenation goals.

### Data collection

At the time of admission, the following parameters were recorded: age, sex, Simplified Acute Physiology Score (SAPS) II [10], Sepsis-related Organ Failure Assessment (SOFA) score [12], reason for admission to the ICU (medical, elective surgery or emergency surgery) and the total bilirubin and creatinine blood levels. On inclusion, the main cause of ARDS, time from intubation to randomization, ventilator settings including the plateau pressure, P/F ratio, SOFA score (with its components), arterial blood gases, arterial blood lactate level, platelets count, and total bilirubin and creatinine blood levels were recorded. The total serum bilirubin was also measured at days 2, 4, 7 and 14.

### Main outcomes evaluated

The main objective of the study was to determine whether early hepatic dysfunction evaluated by the measurement of bilirubin on inclusion was associated with the 90-day mortality rate. The secondary endpoints were the 28-day mortality rate, the time to be weaned from mechanical ventilation and the number of ventilator-free days until day 90 according to the presence or not of an early hepatic dysfunction.

### Definition

Hepatic dysfunction was defined as a serum bilirubin ≥ 33 μmol/L (a SOFA hepatic sub-score ≥ 2).

### Statistical analysis

If not mentioned, the data are expressed as the means with standard deviation for the continuous variables and as the number and percentages for the categorical variables. The survivors and non- survivors were first compared using a univariate analysis (a chi-square test or Student’s t-test). A multivariate logistic Cox analysis was then performed to determine whether an increased bilirubin level was independently associated with the 90-day mortality in the ARDS patients. A post-hoc analysis was done regarding day-90 mortality according to the tertiles of Pa2/FiO2 and bilirubin at inclusion (population divided into three groups from the lowest value to the highest one, each containing a third of the population). All of the variables with a p value < 0.10 were included in the model after excluding the presence of a collinearity. The patient survival by inclusion of the serum level of bilirubin classified according to the SOFA score classes for hepatic sub-score was analysed by the Kaplan-Meier method and compared with the covariance matrix for log-rank statistics (The LIFETEST Procedure of SAS System, NC, USA). If not mentioned, the statistical analysis was conducted using SPSS, version 20.0 (NY, USA).

## Results

### Characteristics of the patients

Table 1 shows the baseline characteristics of the 805 included patients. Most of the patients were admitted for a medical reason (n = 666, 83%). The main cause of ARDS was infectious pneumonia (n = 475, 59%). The mean time between the ICU admission and inclusion was 2.2±3.0 days. The patients were severely hypoxaemic on inclusion, with a P/F ratio of 107±31 mmHg. The serum bilirubin on inclusion was 26±43 μmol/L (S1 file). The incidence of early hepatic dysfunction was therefore of 17.6% (n = 142). The 90-day mortality rate of the entire cohort was 33.8% (n = 272). Concerning other important outcomes, the overall 28-day mortality was 25.6% (n = 206). The duration of mechanical ventilation was 18.5±16.7 days for the entire cohort. The number of ventilator-free days to day 90 for the entire population was 61.5±28.8 days.

**Table 1:** General characteristics between survivors and non survivors.

	All	Survivors	Non-survivors	*P*
	(n = 805)	(n = 533)	(n = 272)	
**Admission**				
**Age (yr)**	59±16	56±15	66±14	<0.0001
**Male sex (no, %)**	556 (69)	366 (69)	190 (70)	0.73
**SOFA score**	9.7±3.5	9.2±3.4	10.7±3.6	<0.0001
**SAPS II score**	47±16	45±17	51±15	<0.0001
**Type of ICU admission (no, %)**				0.009
***medical***	666 (83)	428 (80)	238 (88)	
***surgery elective***	54 (7)	39 (7)	15 (6)	
***surgery emergency***	85 (11)	66 (12)	19 (7)	
**Bilirubin (μmol/l)**	13 [8–24]	12 [8–21]	14 [9–34]	0.002
**Creatinine (μmol/l)**	97 [68–164]	92 [65–152]	120 [78–184]	0.0001
**Inclusion**				
**Main cause of ARDS (no, %)**				0.76
***infectious pneumonia***	475 (59)	307 (57)	168 (62)	
***aspiration pneumonitis***	160 (20)	115 (22)	45 (17)	
***lung contusion***	10 (1)	10 (2)	0 (0)	
***extra-pulmonary sepsis***	66 (8)	44 (8)	22 (8)	
***Others***	94 (12)	57 (11)	37 (14)	
**SOFA score**	10.0±3.3	9.4±3.2	11.1±3.3	<0.0001
**SOFA score w/o liver**	9.5±3.0	9.0±3.0	10.3±2.9	<0.0001
**SOF**				
***Hepatic***	0.55±0.96	0.42±0.81	0.81±1.17	<0.0001
***Respiratory***	3.42±0.52	3.40±0.52	3.45±0.53	0.22
***Cardiovascular***	2.95±1.51	2.77±1.62	3.31±1.20	<0.0001
***Renal***	1.14±1.50	0.95±1.40	1.51±1.63	<0.0001
***Neurological***	1.51±1.53	1.56±1.56	1.43±1.47	0.46
***Coagulation***	0.69±0.96	0.61±0.91	0.83±1.04	0.003
**Tidal volume (mL/kg)**	6.3±0.8	6.3±0.8	6.3±0.9	0.93
**Respiratory rate (breaths/min)**	26±5	26±5	26±5	0.19
**Plateau pressure (cm H**_**2**_ **O)**	24±5	24±5	25±5	0.06
**PEEP applied (cm H**_**2**_ **O)**	10±3	10±3	10±4	0.58
**Total PEEP (cm H**_**2**_ **O)**	11±5	11±4	11±7	0.52
**FiO**_**2**_	0.79±0.17	0.78±0.17	0.79±18	0.38
**P/F ratio**	107±31	107±31	105±32	0.35
**Arterial pH**	7.31±0.10	7.33±0.09	7.28±0.12	<0.0001
**PaO**_**2**_ **(mmHg)**	81±22	81±21	80±22	0.74
**PaCO**_**2**_ **(mmHg)**	49±20	48±11	51±31	0.024
**Lactate (mmol/L)**	2.4±2.9	2.2±2.8	2.9±3.0	0.001
**Bilirubin (μmol/l)**	12 [7–23]	11 [7–20]	13 [8–35]	0.001
**Creatinine (μmol/l)**	96 [67–162]	89 [63–152]	113 [77–179]	0.0001

Quantitative values are expressed as means ± SD or median [IQR] and qualitative values as numbers (percentage); ICU, intensive care unit; SAPS II, Simplified Acute Physiology Score II; SOFA, Sequential Organ Failure Assessment; ARDS, acute respiratory distress syndrome; PEEP, positive end-expiratory pressure; FiO_2_, fraction of inspired oxygen; P/F ratio, the ratio of the partial pressure of arterial oxygen to the fraction of inspired oxygen; PaO_2_, partial pressure of arterial oxygen; PaCO_2_, partial pressure of arterial carbon dioxide

### Evolution of bilirubin

**Fig 1:**
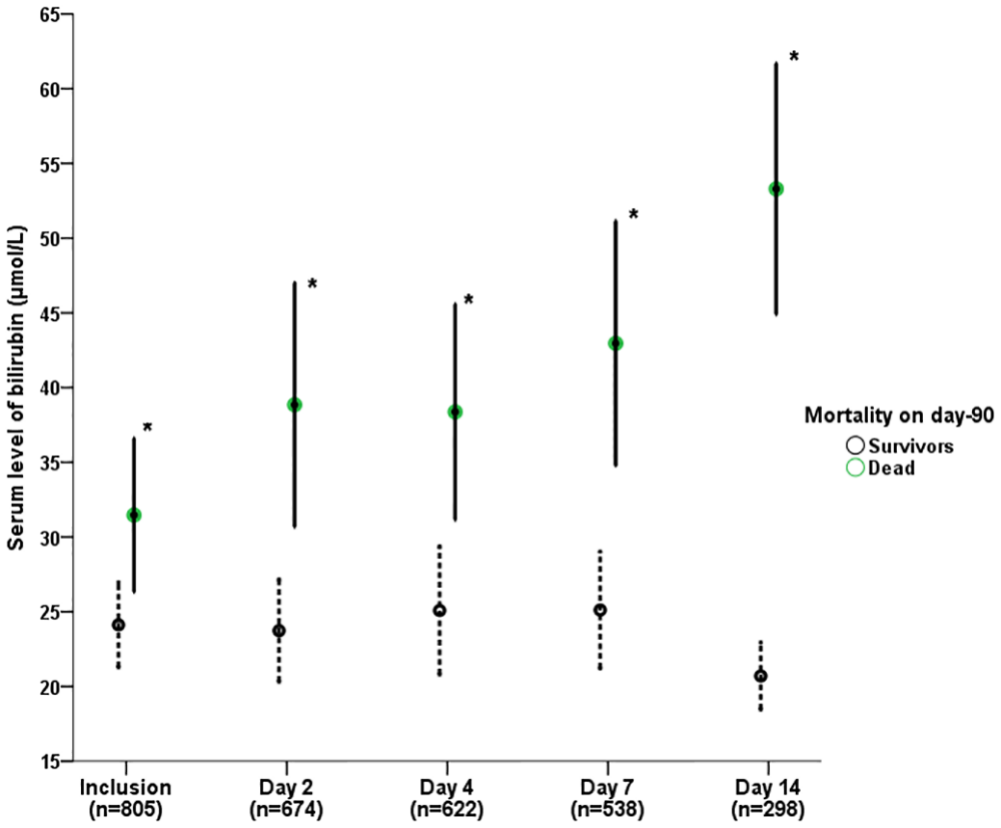
Evolution of serum bilirubin level in survivors and non survivors. Mean ± SEM. *: p value <0.001 (survivors vs. non-survivors).

The changes in the serum bilirubin levels in the survivors and non-survivors are presented in Fig 1. The serum bilirubin levels in the non-survivors were persistently higher than in the survivors from inclusion to the end of the observational period (p<0.0001). When the effect of prone positioning was evaluated in the group of patients included in the PROSEVA study, there was no effect of this strategy on bilirubin levels (data not shown).

### Early hepatic dysfunction and outcomes

**Fig 2:**
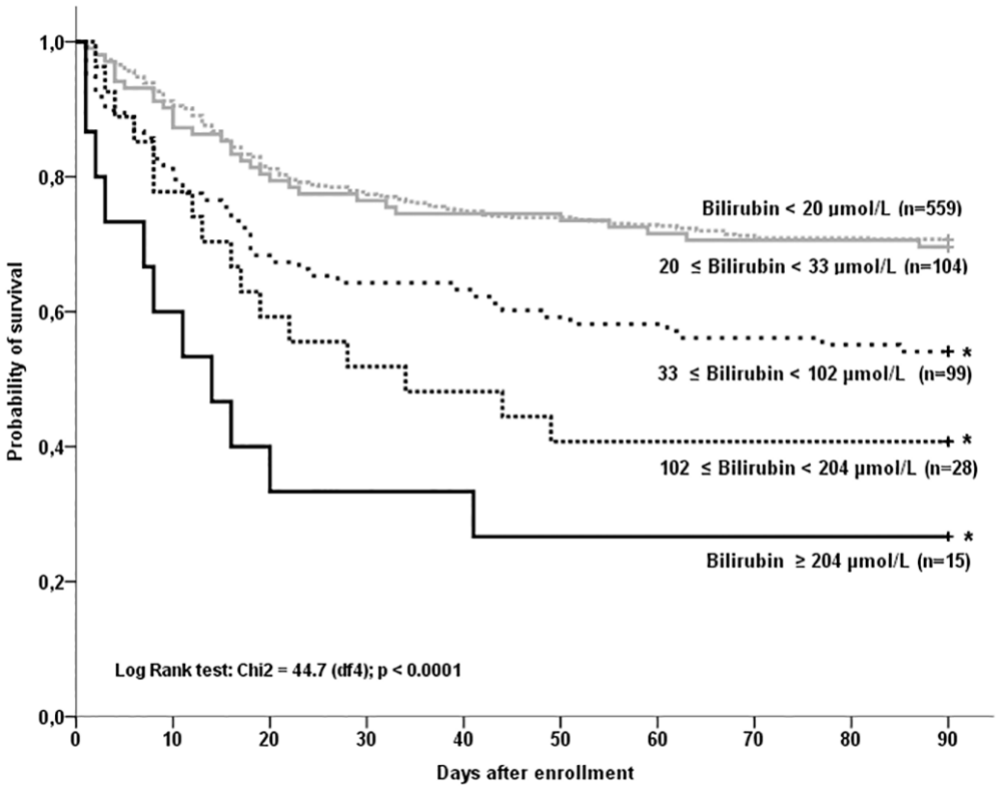
Probability of survival in ARDS patients according to the bilirubin level at inclusion. *: p <0.001 (comparisons between each strata of serum bilirubin level, bilirubin < 20 μmol/L was used as the reference curve).

A Kaplan-Meier survival analysis shows that the patients with a serum bilirubin ≥ 33 μmol/L on inclusion had a significantly lower survival rate than did patients with serum bilirubin level < 33 μmol/L (Fig 2). Using this threshold, sensitivity to predict death at day 90 was low (27%, 95% confidence interval from 22% to 33%), as positive predictive value (52%, 95% confidence interval from 44% to 60%). In contrast specificity was good (87%, 95% confidence interval from 84% to 90%). Negative predictive value was 70% (95% confidence interval from 66% to 74%). The 90-day mortality rate was 29.9% (n = 198) for the patients without hepatic dysfunction and 52.1% (n = 74) for the patients with hepatic dysfunction (p<0.0001). The mortality at day 28 was 22.2% (n = 147) for the patients without early hepatic dysfunction and 41.5% (n = 59) for those with early hepatic dysfunction (p<0.0001). The duration of mechanical ventilation was 18.7±15.9 days for the patients without early hepatic dysfunction as compared with 17.9±20.2 days for the patients with early hepatic dysfunction (p = 0.65). The number of ventilator-free days to day 90 was decreased in the group of patients with early hepatic dysfunction (55.9±34.2 days vs. 62.7±27.4 days for the patients without early hepatic dysfunction; p = 0.027).

### Factors associated with death at day 90

#### Univariate analysis

As shown in Table 1, the deceased patients were older and had higher SOFA and SAPS II scores at admission. The serum bilirubin was higher in the non-survivors than in the survivors at admission and upon inclusion (Table 1). The non-survivors had also a lower arterial pH level and higher serum lactate levels. The hepatic, cardiovascular, renal and coagulation components of the SOFA score on inclusion were higher in the non-survivors than in the survivors. In contrast, there were no significant differences between the survivors and non-survivors regarding the principal cause of ARDS, the mechanical ventilation parameters and the P/F ratio. There was no correlation between PaO2/FiO2 ratio and bilirubin on inclusion (data not shown). As expressed in Table 2, day-90 mortality increased when bilirubin increased, while it remained unchanged even for the 33% of the patients exhibiting the most profound hypoxaemia (1^st^ tertile).

**Table 2:** Mortality at Day 90 according to tertiles of PaO2/FiO2 and bilirubin on inclusion.

	Day-90 mortality			*P*
	1^st^ tertile	2^nd^ tertile	3^rd^ tertile	
**PaO2/FiO2**	36.9%	32.1%	32.5%	NS
**Bilirubin**	29.8%	28.7%	41.5%	0.002

#### Multivariate analysis

The following variables on inclusion were included in the Cox model: age, the hepatic SOFA score, the cardiovascular SOFA score, the renal SOFA score, the coagulation SOFA score, the arterial pH level and the plateau pressure. Three variables (PaCO_2_, creatinine and the arterial lactate levels) were discarded because they presented collinearity. As shown in Table 3, the multivariate Cox regression analysis identified the age, the hepatic SOFA score, the coagulation SOFA score, the arterial pH level, and the plateau pressure as being independently associated with 90-day mortality in patients with ARDS. A bilirubin of 33 μmol/L or more is independently associated with 90-day mortality (Hazard ratio 2.32; 95% confidence interval from 1.74 to 3.09; p <0.0001).

**Table 3:** Multivariate Cox regression analysis for factors associated with ARDS mortality at Day 90.

	Hazard Ratio (95% CI)	*P*
**Age (per year)**	1.05 (1.04–1.06)	<0.0001
**SOFA Hepatic score (per unit)**	1.43 (1.28–1.61)	<0.0001
**SOFA Cardiovascular score (per unit)**	1.06 (0.96–1.18)	0.24
**SOFA Renal score (per unit)**	1.05 (0.97–1.14)	0.26
**SOFA Coagulation score (per unit)**	1.16 (1.03–1.32)	0.019
**pH inclusion (per unit)**	0.06(0.02–0.21)	<0.0001
**Plateau pressure, cm H2O (per cm H2O)**	1.03 (1.00–1.06)	0.037

The HR and 95% CI were calculated using a multivariate Cox proportional hazard model

## Discussion

The results of the present study suggested that the presence of a hepatic dysfunction during the first 48-h period of moderate-to-severe ARDS is strongly associated with the outcome. Despite a better understanding of the pathophysiology of lung injury and improved management of patients with ARDS, the mortality rate remains high. It is generally admitted that the deaths occurring early in the course of ARDS are mainly associated with a refractory hypoxemia, late deaths being associated with a progressive multiple organ failure. The results of the present study suggested that organs other than lung are also injured early in the course of ARDS. It also indicated that early liver dysfunction and not kidney dysfunction is independently associated with death in ARDS patients ventilated according to a protective ventilation strategy.

Few studies have suggested that serum bilirubin could be an early and sensitive biomarker of ARDS development and mortality in sepsis. In 1989, Schwartz et al. [15] reported that the serum bilirubin level was significantly lower in survivors than in non-survivors on the first day of ARDS in 24 ARDS patients. In this latter series [15], mortality was high at 58% and patients were ventilated on inclusion with a low PEEP level. Interestingly, neither oxygenation nor renal function differentiated survivors from non-survivors [15]. More recently Zhai et al. [8] showed that increased bilirubin on admission was associated with a higher day-60 mortality rate in a selected population of sepsis-related ARDS patients from a single-center study. The same group confirmed that admission bilirubin was independently associated with ARDS mortality [7]. There was no information regarding the treatment of ARDS (mechanical ventilation mode, prone positon, PEEP strategy, tidal volume) [7]. These results suggest that increased serum bilirubin on ICU admission could also be a good surrogate of mortality in ARDS patients. However, the present study is to the best of our knowledge the first to show that serum bilirubin evaluated on ARDS onset is associated with day-90 mortality in a series of patients ventilated according to a lung protective ventilation strategy and included in interventional trials using well-defined oxygenation goals and criteria of severe ARDS with a P/F ratio lower than 150. Additional studies have investigated the incidence of liver dysfunction and its effect on the outcome of critically ill patients [5, 6, 16]. Finally, liver failure occurs frequently during the ICU stay [16] and is strongly related to ICU mortality [5].

From a physiopathological point of view, ARDS is associated with an increased microvascular permeability resulting in pulmonary edema. This microvascular injury could also involve the hepatic microvascular circulation [17–19]. The hepatic damages appear to be granulocyte mediated, as described in the lung [20]. Liver dysfunction might result from vascular damage and/or edema. Liver injury can be implied in the development of other organ failures (such as the lung) and subsequent mortality. Hepatocellular damage could increase the hepatic synthesis of a substance that potentiates acute lung injury or interferes with other organs functions. Bilirubin is able to induce inflammation, apoptosis and oxidative stress [21–27]. Bilirubin at high levels could be therefore an active participant in the maintenance of ARDS and its related worse outcome.

In patients with ARDS in the prone position, we wondered whether this position could have an effect on the hepato-splanchnic circulation. We compared the serum bilirubin levels in patients with ARDS in the prone and supine position only in the patients included in the PROSEVA study. Indeed, the use of prone positioning in the ACURASYS study was left to the discretion of the clinicians. We found that there was no significant difference between the groups in the PROSEVA study (data not shown), which is in agreement with the study of Matejovic et al. [28], who showed in a small series of 11 patients with acute lung injury that prone positioning did not affect the intra-abdominal pressure or the hepato-splanchnic blood flow nor splanchnic oxygen consumption. We cannot exclude the fact that some drugs could have altered the bilirubin levels of patients included in the present study. However, Brienza et al. [29] showed no deleterious effects of 17 potentially hepatotoxic drugs when administered to ICU patients. The increase of the bilirubin level at admission in this study could be explained by the use of vasopressors as well. The principal response of the hepatic vascular bed to catecholamines is vasoconstriction [30, 31]. Norepinephrine and epinephrine divert blood flow from the mesenteric circulation and decrease the microcirculatory blood flow in the gastro-intestinal tract, despite increasing perfusion pressure and systemic blood flow in experimental sepsis [32]. However, we did not identify any difference regarding vasopressor use in this study, as assessed by the SOFA score. While bilirubin is usually measured to investigate liver function in ICU patients, it has been recently suggested that this diagnostic approach could underestimate the problem [17]. In contrast plasma bile acids measurement seems attractive in sepsis by early indicating liver dysfunction and by presenting a higher sensitivity and specificity as compared with bilirubin in predicting death at day 28 [33]. However, more clinical investigations are needed to confirm these results. In the present study, no other parameter was evaluated in order to assess liver function.

## Conclusion

Hepatic dysfunction is observed early in the course of ARDS patients and is associated with the 90-day mortality rate. Future research are needed to elucidate the potential pathogenic role of bilirubin in ARDS.

## Supporting Information

S1 File Extract database biliards. (XLSX)
